# Traffic signal coordination control along oversaturated two-way arterials

**DOI:** 10.7717/peerj-cs.319

**Published:** 2020-11-23

**Authors:** Haitao Xu, Zuozhang Zhuo, Jing Chen, Xujian Fang

**Affiliations:** School of Computer Science and Technology, Hangzhou Dianzi University, Hangzhou, Zhejiang, China

**Keywords:** Traffic congestion, Traffic signal coordination control, Traffic demand, Two-way arterials

## Abstract

As an effective method to alleviate traffic congestion, traffic signal coordination control has been applied in many cities to manage queues and to regulate traffic flow under oversaturated traffic condition. However, the previous methods are usually based on two hypotheses. One is that traffic demand is constant. The other assumes that the velocity of vehicle is immutable when entering the downstream section. In the paper, we develop a novel traffic coordination control method to control the traffic flow along oversaturated two-way arterials without both these hypotheses. The method includes two modules: intersection coordination control and arterial coordination control. The green time plan for all intersections can be obtained by the module of intersection coordination control. The module of arterial coordination control can optimize offset plan for all intersections along oversaturated two-way arterials. The experiment results verify that the proposed method can effectively control the queue length under the oversaturated traffic state. In addition, the delay in this method can be decreased by 5.4% compared with the existing delay minimization method and 13.6% compared with the traffic coordination control method without offset optimization. Finally, the proposed method can balance the delay level of different links along oversaturated arterial, which can directly reflect the efficiency of the proposed method on the traffic coordination control under oversaturated traffic condition.

## Introduction

With the increase of traffic demand in urban area, traffic congestion problems during peak hours are more and more severe (Li & Schonfeld, 2015). Traffic signal coordination control as an effective traffic control approach has been applied in many cities to alleviate traffic congestion. Arterial as the important holder of urban traffic is the primary object of traffic coordination control. How to improve the traffic coordination control method along arterials to promote the efficiency of traffic control has attracted great attention recently.

According to the optimization objective, traffic coordination control method can be classified into two types (Luo & Zhang, 2019): bandwidth maximization method and performance optimization method. On bandwidth maximization method, Little (1966) proposed the classic maxband model for the first time to coordinate a two-way arterial. To model the traffic in reality, Little, Kelson & Gartner (1981) took into consideration the impact of queue on traffic model. However, the improved maxband model assumed the bandwidth was constant. To deal with the above restriction, Gartner et al. (1991) proposed the multiband model, which can assign different weights to different links to adapt to unbalanced distribution of real traffic flow. To conquer the limitation of symmetry properties of the above model, the am-band (Zhang, Xie & Gartner, 2015) model was proposed. On the performance optimization method, Hillier & Rothery (1967) first proposed the delay minimization method to optimize the offset of arterial with two intersections. However, this method is based on the delay along one-way arterials. Therefore, a two-way delay triangle model for two-way is proposed (Lieberman & Woo, 1976).

Although the aforementioned traffic coordination control method can improve traffic signal control to a certain extent, when the arterial is under oversaturated traffic condition, the traffic control effect is going to be poor. Therefore, the research on traffic coordination control along oversaturated arterial has attracted much attention. The traffic coordination control method along oversaturated arterial should ensure performance optimization and queue length minimization simultaneously. Lu, Liu & Dai (2008) improved the travel time distribution function in the MAXBAND model by analyzing the discrete phenomenon of vehicles under oversaturated traffic condition to obtain optimal traffic signal control coordination parameters. Qi (2017) proposed an improved graphical method with the queue length estimated by the vehicle queuing accumulation curve to control the queue spillover along oversaturated arterials. Abu-Lebdeh & Benekohal (2000) proposed a two-way coordination control method, which can limit the vehicle queue to avoid the spillover to the links of upstream intersection. Gazis (1964) proposed an improved delay minimization method to coordinate the oversaturated arterial with two adjacent intersections. However, this method only took the performance index of total delay into account, which cannot control the queue length under oversaturated traffic condition effectively. To solve this problem. Ramezani & De Lamberterie (2016) proposed a novel method is to implement a real-time partitioning of the arterial to detect critical cluster(s) of consecutive links with oversaturated traffic conditions, control arterial queue length spillbacks. To alleviate the queue spillovers at intersections of urban arterials, Ren et al. (2018) proposed a novel method to calculating overflow tendency towards intersection queuing, can better prevent the overflow of queues at arterials intersections. Ramezani et al. (2015) proposed a traffic signal control method in congested urban arterials by integrating a clustering approach into a control policy of long queues prior to spillover occurrence to reduce the risk of spillovers through a feedback strategy. He, Kamineni & Zhang (2016) proposed grade separation at signalized intersections to optimize signal time at all intersections along oversaturated arterials. Hu, Wu & Liu (2013) analyzed the detrimental impacts of residual queue and queue spillover to manage oversaturated arterials. Ramezani et al. (2017) proposed a real-time adaptive signal control method to avoid spillover along oversaturated arterial. In view of the fact that the arrival rate of vehicles may change over time, the existing research neglected the randomness of vehicles arrival. Therefore, an adaptive coordination control method (Hua et al., 2015; Tong et al., 2015; Li, Huang & Lo, 2018) which considered the randomness of traffic was proposed to optimize the traffic coordination control on oversaturated arterials. In this paper, we propose a novel traffic signal coordination method which includes two modules: intersection coordination control and arterial coordination control. The details of the contributions in this paper include three aspects which can be listed as below:

(1) Intersection coordination control method based on model predictive control is applied to obtain the optimal green time scheme.

(2) Considering the vehicle speed is changing when entering the tail of queue, we propose an improved delay minimization model to model the traffic evolution in reality. Then, the proposed IGAABC algorithm is used to solve the optimal coordination parameters. Finally, the modules of arterial spillover detection and management can be used to avoid spillover along oversaturated arterial.

(3) Considering the randomness of vehicles arrival, the rate of vehicles arrival can be estimated based on prediction model to modify the improved delay minimization model.

The rest of the paper is organized as follows. The ‘Method Framework’ section describes the control framework of the proposed method. The ‘Intersection Coordination Control’ section introduces the module of intersection coordination control. The arterial coordination control method is given in ‘Arterial Coordination Control’, which contains three parts: offset optimization, arterial spillover detection and arterial spillover management. We discuss the comparison results of simulation evaluation in ‘Case Study’. Conclusions and future research are put forward in ‘Conclusion and future outlook’.

## Method Framework

**Figure 1:**
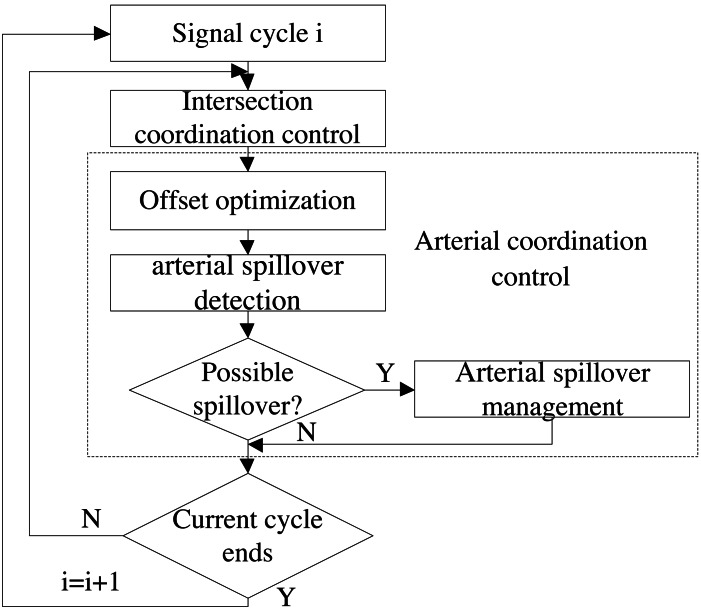
Framework of the traffic coordination control method.

Traffic coordination control method presented in this paper, mainly contains two parts: intersection coordination control and arterial coordination control. Intersection coordination control can be applied to optimize green time plans for all intersections along oversaturated arterials. As shown in Fig. 1, arterial coordination control is composed of three modules: offset optimization, arterial spillover detection and arterial spillover management, which can optimize the parameters of offset for all intersections and manage queues simultaneously.

### Intersection coordination control

The proposed intersection coordination control method is based on model predictive control, which consists of prediction model, optimization model and optimization algorithm (Xu, Chen & Xu, 2019; Zhou et al., 2017). At the beginning of each cycle, the prediction model will first predict traffic information according to collected real-time number of vehicles and queue length for each link. And then, it takes travel time as the optimization objective to construct optimization model according to predicted traffic information. Finally, IGAABC is used to solve optimization model to obtain optimal green time plans for all intersections.

### Arterial coordination control

According to the optimal green time plans obtained by intersection coordination control, the module of offset optimization can construct delay model along oversaturated arterials to obtain optimal offset which is solved by IGAABC algorithm. Then the basic control sequence of green time and offset plans for all intersections can be applied to a simulated arterial. Afterwards, arterial spillover detection module will detect potential spillover for all links along oversaturated arterial according to the real-time speed and the vehicle count. If queue overflow is detected at one of the phases corresponding to the same signal group, the module of arterial spillover management can be activated to avoid spillover of the corresponding signal group. Otherwise, it will be decided whether the current control cycle is completed. If yes, the next loop continues. Otherwise, arterial spillover detection module will continue to identify potential spillover.

## Intersection Coordination Control

Green time plans as important coordination parameter can determine how many vehicles can be released for each intersection. In this paper, intersection coordination control, which is based on model predictive control strategy (Zhou et al., 2017), is applied to optimize green time plans. The algorithmic steps will be introduced next.

At the beginning of each control moment, prediction model will collect the information of queue length and vehicle count for each link along oversaturated arterial by the corresponding detection. Subsequently, prediction model will be applied to predict vehicle count over several control time steps according to real-time traffic information. After that, total travel time cost is selected as the optimization objective to optimize the green time plans for each intersection. Finally, IGAABC algorithm is applied to solve the optimal green time plans. A detailed description of prediction model, optimization model and IGAABC optimization algorithm has been provided in our previous article (Xu, Chen & Xu, 2019).

## Arterial Coordination Control

Intersection coordination control provides optimal green time plans, which can optimize local traffic control. However, the interaction between intersections along oversaturated arterials, where the traffic flow conditions on each road segment will affect each other, cannot be neglected. Therefore, arterial coordination control is presented to make sure that vehicles can continuously pass through several intersections along oversaturated arterials as many as possible. A large number of scholars have studied arterial coordination control method. There are two main categories: bandwidth maximization method and performance optimization method. These two kinds of methods are able to work effectively when the traffic flow is low. However, with the increase of traffic density, the control effect of these methods gets worse and worse. To optimize the coordination parameter of offset, we propose an improved delay minimization method. We first take into consideration the evolution of queuing under oversaturated arterials. In addition, we analyze how traffic density affects vehicle speed when the traffic flow enters the downstream section and modify the delay model. Finally, the rate of traffic arrival can be predicted according to the model predictive control, which can adapt to the situation that traffic demand is not constant. To control queue along oversaturated arterials, we adopt the strategy of arterial spillover detection and management. In the following section, we will introduce offset optimization, arterial spillover detection and arterial spillover management separately.

### Offset optimization

#### Delay analysis for oversaturated arterial

Considering that the law of traffic flow evolution of oversaturated arterial with multiple intersections is similar to the arterial with two adjacent intersections, we take the arterial with two adjacent intersections as an example to analyze delay model. The relationship between delay and coordination control parameters can be classified as two types (Li, Huang & Lo, 2018). The first type of delay, called general delay, represents the delay at the entrance of the first intersection along oversaturated arterial and the delay for every secondary section. The arrival pattern of traffic flow cannot be affected by the upstream intersection for this kind of delay.

**Figure 2:**
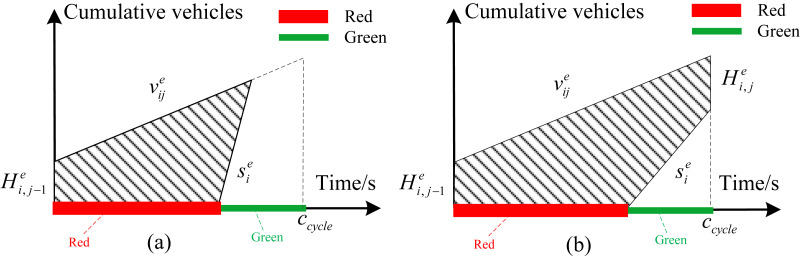
(A) The queue length can be completely eliminated at current cycle; (B) the queue length cannot be completely eliminated during the green time at current cycle.

Figure 2 shows the formation of the first type of delay. ${v}_{ij}^{e}$ represents the arrival rate of traffic flow at phase *i* of intersection *E*^*e*^. In view of that delay is defined as the additional time compared with the normal driving conditions, the total delay of this phase in each cycle can be obtained from the area of the shaded area shown in Fig. 2. ${s}_{i}^{e}$ is saturated flow rate of phase $i.{H}_{i,j}^{e}$ represents the remaining queue length of phase *i* at intersection *E*^*e*^ for cycle *j*. Figure 2A shows the situation where the queuing can be completely eliminated at current cycle. Figure 2B shows the situation where queue length cannot be completely eliminated during the green time at current cycle. Equation (1) represents the delay calculation formula in this case. We define $RA={c}_{cycle}-{G}_{i}^{e}$ and $T{H}_{i,j}^{e}={H}_{i,j-1}^{e}+{v}_{ij}^{e}\cdot {c}_{cycle}$.

(1) \begin{eqnarray*}{d}_{ij}^{e}= \left\{ \begin{array}{@{}l@{}} \displaystyle \frac{1}{2\cdot {c}_{cycle}\cdot {v}_{ij}^{e}} \cdot \frac{{s}_{i}^{e}\cdot RA \left[ 2{H}_{i,j-1}^{e}+{v}_{ij}^{e}\cdot RA \right] +{ \left( {H}_{i,j-1}^{e} \right) }^{2}}{{s}_{i}^{e}-{v}_{ij}^{e}} ,{G}_{i}^{e}\cdot {s}_{i}^{e}\gt T{H}_{i,j}^{e}\\ \displaystyle \frac{1}{2\cdot {c}_{cycle}\cdot {v}_{ij}^{e}} \cdot \left[ {c}_{cycle} \left( 2{H}_{i,j-1}^{e}+{v}_{ij}^{e}\cdot {c}_{cycle} \right) -{ \left( {G}_{i}^{e} \right) }^{2}{s}_{i}^{e} \right] ,{G}_{i}^{e}\cdot {s}_{i}^{e}\leq T{H}_{i,j}^{e} \end{array} \right. \end{eqnarray*}

**Figure 3:**
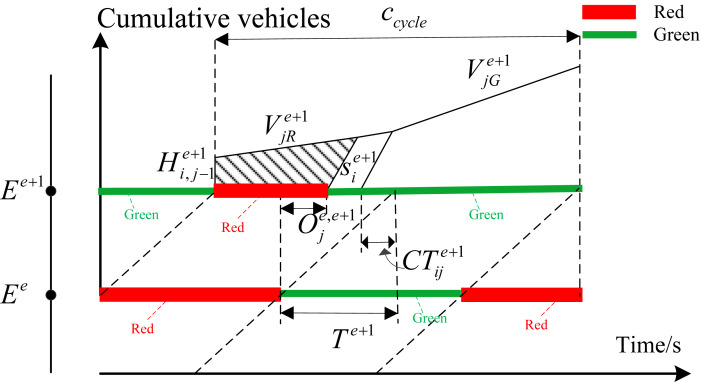
Delay relationship with enough green time.

The second type of delay is mainly for interrelated intersections called interrelated delay. Figure 3 depicts the evolution of traffic flow from upstream intersection *E*^*e*^ to downstream intersection *E*^*e*^. The number of vehicles arriving at the intersection during the red time of coordinated phase *i* from intersection *E*^*e*^ to intersection *E*^*e*+1^ depends on the turning vehicle leaving from secondary section. The traffic arrival rate during red time is relatively small in this case. The arrival rate during green time contains vehicles traveling straight along coordinated direction and vehicles turning from secondary section. The total number of vehicles arriving at intersection *E*^*e*+1^ in the *j*th cycle for phase *i* can be expressed as $T{A}_{ij}^{e+1}={v}_{jG}^{e+1}\cdot {G}_{i}^{e}+{v}_{jR}^{e+1}\cdot {R}_{i}^{e}$. Obviously, the vehicle amount of traffic arriving at intersection *E*^*e*+1^ depends on the arrival rate of vehicles along the coordinated direction and the green time plan of upstream intersection. We define CT as the remaining queue clearing time. The remaining queue clearing time in the *j*th cycle for phase *i*can be obtained according to the equation of $C{T}_{ij}^{e+1}=({H}_{i,j-1}^{e+1}+{v}_{jR}^{e+1}\cdot {R}_{i}^{e})/{s}_{i}^{e+1}.{T}^{e+1}$ isdefined as the time it takes for the vehicle to merge from the parking line at the upstream intersection into the entrance of the downstream section. The equation of delay calculation with different offset is different. Therefore, the delay expression can be defined according to the actual situation as follows:

(1) When the green time ${G}_{i}^{e+1}$of downstream intersection *E*^*e*+1^ is long enough to meet the equation of $C{T}_{ij}^{e+1}+{G}_{i}^{e}\leq {G}_{i}^{e+1}$, the delay can be denoted by the shaded area in Fig. 3.

As shown in Eq. (2), the relationship between delay and offset in this case can be determined according to the geometric relationship. Define the red time at the intersection *E*^*e*+1^ as $RB={c}_{cycle}-{G}_{i}^{e}-{T}^{e+1}+{O}_{j}^{e,e+1}$.

**Figure 4:**
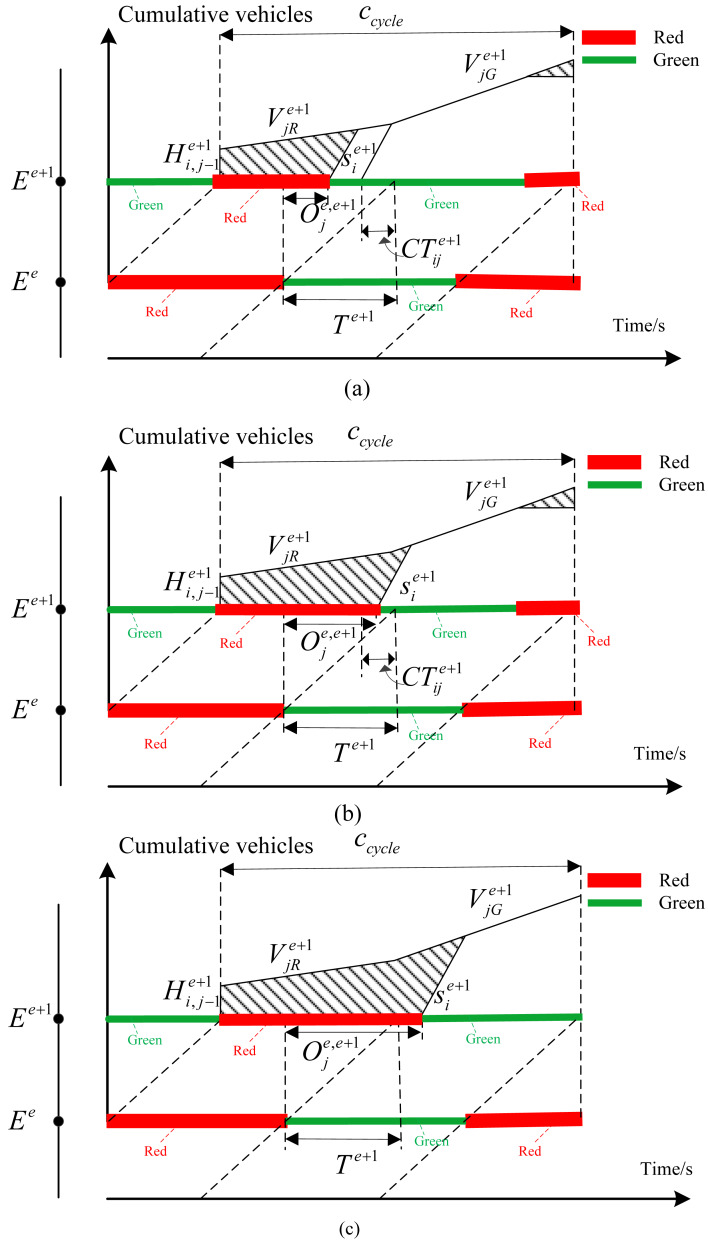
(A) The remaining queues in the previous cycle can be cleared, but the green time is not enough for all vehicles to pass through the intersection; (B) when the red time and the traffic queue are over, they cannot completely pass through the intersection; (C) the last cycle remaining queue and red time cannot pass, but the green time can pass the intersection.

(2) \begin{eqnarray*}{d}_{ij}^{e+1}= \frac{1}{2T{A}_{ij}^{e+1}} \left[ \frac{{s}_{i}^{e+1}\cdot RB \left[ 2{H}_{i,j-1}^{e+1}+{v}_{jR}^{e+1}\cdot RB \right] +{ \left( {H}_{i,j-1}^{e+1} \right) }^{2}}{{s}_{i}^{e+1}-{v}_{jR}^{e+1}} \right] \end{eqnarray*}

(2) The green time ${G}_{i}^{e+1}$ meet the inequality relationship of $C{T}_{ij}^{e+1}\lt {G}_{i}^{e+1}\leq C{T}_{ij}^{e+1}+{G}_{i}^{e}$. In this case, the green time of downstream intersection *E*^*e*+1^ isenough to clear the remaining queue in last cycle. However, it cannot be guaranteed that all vehicles that arrive at intersection *E*^*e*+1^ during green time from the upstream intersection can pass completely during the green time of intersection *E*^*e*+1^. Delay is different with different offset. Therefore, the delay can be classified as three types according to the relationship between offset and green time. The corresponding delay can be shown in Figs. 4A–4C.

As shown in Fig. 4A, the offset meets the inequality relationship of $0\leq {O}_{j}^{e,e+1}\lt {T}^{e+1}-C{T}_{j}^{e+1}$, which represents the offset is small. When the vehicles from the secondary road of upstream intersection *E*^*e*^reach the intersection *E*^*e*+1^, the remaining queue length of last cycle can be completely emptied. However, the remaining green time is too small to allow all vehicles at the end of the traffic queue to pass through the intersection *E*^*e*+1^, resulting in increased delay in this direction. According to the geometric relationship, the delay relationship in this case is shown in Eq. (3).

(3) \begin{eqnarray*}{d}_{ij}^{e}= \frac{1}{2T{A}_{ij}^{e+1}} \left[ \frac{{s}_{i}^{e+1}\cdot RB \left[ 2{H}_{i,j-1}^{e+1}+{v}_{jR}^{e+1}\cdot RB \right] +{ \left( {H}_{i,j-1}^{e+1} \right) }^{2}}{{s}_{i}^{e+1}-{v}_{jR}^{e+1}} +{ \left( {c}_{cycle}-RB \right) }^{2}\cdot {v}_{jG}^{e+1} \right] \end{eqnarray*}

As shown in Fig. 4B, with the increase of coordination parameter of offset, which meets the inequality relationship of ${T}^{e+1}-C{T}_{j}^{e+1}\leq {O}_{j}^{e,e+1}\leq {T}^{e+1}$. The traffic flow from the secondary road during the red time of the upstream intersection cannot completely pass through the intersection, which increases delay. In addition, vehicles which cannot pass through the intersection at the end of the traffic queue have also caused the increase of delay. In this case, the delay is composed of these two parts. According to the geometric relationship, the delay can be calculated according to Eq. (4).

(4) \begin{eqnarray*}{d}_{ij}^{e}= \frac{1}{2T{A}_{ij}^{e+1}} \left[ \begin{array}{@{}l@{}} \displaystyle RA \left[ {v}_{jR}^{e+1}\cdot RA+2{H}_{i,j-1}^{e+1} \right] -{s}_{i}^{e+1}{ \left( {T}^{e+1}-{O}_{j}^{e,e+1} \right) }^{2}+{ \left( {c}_{cycle}-RB \right) }^{2}\cdot {v}_{jG}^{e+1}\\ \displaystyle + \frac{{s}_{i}^{e+1} \left[ {v}_{jR}^{e+1}\cdot RA+{H}_{i,j-1}^{e+1}-{s}_{i}^{e+1}{ \left( {T}^{e+1}-{O}_{j}^{e,e+1} \right) }^{2} \right] }{{s}_{i}^{e+1}-{v}_{jG}^{e+1}} \end{array} \right] \end{eqnarray*}

The offset continues to increase to the range of ${T}^{e+1}\leq {O}_{j}^{e,e+1}\lt {T}^{e+1}+{G}_{i}^{e}-{G}_{i}^{e+1}$, as shown in Fig. 4C. The remaining queue in the previous cycle and the traffic flows entering from the secondary road of upstream intersection during red time cannot pass through the downstream intersection. However, the green time is enough to allow all queued vehicles to pass so that there will be no delay at the end of the traffic queue. In this case, the delay formula is shown in Eq. (5).

(5) \begin{eqnarray*}{d}_{ij}^{e}= \frac{1}{2T{A}_{ij}^{e+1}} \left[ \begin{array}{@{}l@{}} \displaystyle \frac{ \left\{ \begin{array}{@{}l@{}} \displaystyle {v}_{jR}^{e+1,k}\cdot RA+{ \left( {H}_{i,j-1}^{e+1,k} \right) }^{2}+{s}_{i}^{e+1}\cdot \left( {O}_{j}^{e,e+1}-{T}^{e+1} \right) \\ \displaystyle \left[ \left( {O}_{j}^{e,e+1}-{T}^{e+1} \right) \cdot {v}_{jG}^{e+1}+2{v}_{jR}^{e+1}\cdot RA+2{H}_{i,j-1}^{e+1} \right] \\ \displaystyle + \left[ {v}_{jR}^{e+1} \left( {c}_{cycle}-{G}_{i}^{e} \right) +2{H}_{i,j-1}^{e+1} \right] \cdot RA\cdot \left( {s}_{i}^{e+1}-{v}_{jG}^{e+1} \right) \end{array} \right\} }{{s}_{i}^{e+1}-{v}_{jG}^{e+1}} \\ \displaystyle +{ \left( {G}_{i}^{e}-{G}_{i}^{e+1}+{T}^{e+1}-{O}_{j}^{e,e+1} \right) }^{2}\cdot {v}_{jG}^{e+1} \end{array} \right] .\end{eqnarray*}

**Figure 5:**
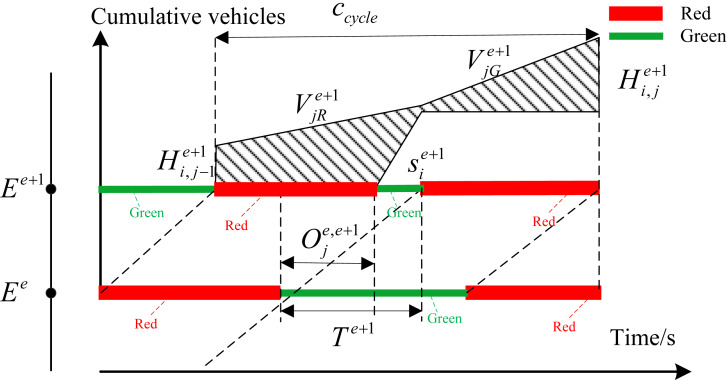
Delay relationship with insufficient green time.

When the green time of the coordination phase of the downstream intersection is too small to eliminate the remaining queue, the delay relationship can be shown in Fig. 5.

In this case, the delay expression can be obtained as shown in Eq. (6).

(6) \begin{eqnarray*}{d}_{ij}^{e+1}= \frac{1}{2T{A}_{ij}^{e+1}} \left[ \begin{array}{@{}l@{}} \displaystyle \left( {c}_{cycle}-{G}_{i}^{e} \right) \left[ {v}_{jR}^{e+1} \left( {c}_{cycle}+{G}_{i}^{e} \right) +2{H}_{i,j-1}^{e+1} \right] \\ \displaystyle +{G}_{i}^{e} \left( {G}_{i}^{e}\cdot {v}_{jG}^{e+1}+2{H}_{i,j-1}^{e+1}+2{v}_{jR}^{e+1}\cdot \left( {c}_{cycle}+{G}_{i}^{e} \right) \right) \\ \displaystyle -{s}_{i}^{e+1}\cdot {G}_{i}^{e+1} \left[ 2{G}_{i}^{e}+{T}^{e+1}-{O}_{j}^{e+1,k}-{G}_{i}^{e+1} \right] . \end{array} \right] .\end{eqnarray*}

The remaining queue length ${H}_{i,j-1}^{e+1}$ in the previous cycle can be obtained by the queue counter. The parameter *c*_*cycle*_ is the signal control cycle time. ${s}_{i}^{e+1}$ is saturation flow rate which can be given directly. Considering that the traffic demand is not constant, the arrival flow rate of ${v}_{ij}^{e},{V}_{jR}^{e+1}$ and ${V}_{jG}^{e+1}$ can be predicted by the prediction model (Xu, Chen & Xu, 2019). Suppose that the distance between the intersection *E*^*e*^ and the intersection *E*^*e*+1^ is *L*^*e*+1^. The driving speed of the vehicle is *v*, the time required for the vehicle to merge from the parking line at the upstream intersection into the entrance of the downstream road can be expressed by Eq. (7).

(7) \begin{eqnarray*}{T}^{e+1}= \frac{{L}^{e+1}}{v} .\end{eqnarray*}

Existing research assumes that the traffic flow is driving with a constant free flow speed into the entrance of the downstream road section. However, the fact is that the speed of the inflow will change according to the traffic flow density. If the traffic flow density is high, the inflow speed will decrease. When the vehicle flow density is low, the speed will increase (Boyles & Ruiz Juri, 2019). Therefore, the expression of speed *v*[*j*] can be defined according to this principle so that the value of can be modified according to the change of traffic flow density.

When the traffic density is less than a certain density threshold *ρ*^min^, which represents that the traffic density is low, the inflow speed of the traffic can be denoted by the free flow speed *v*^*free*^. When the traffic density is larger than the congestion density *ρ*^*jam*^, the traffic can only enter the downstream intersection at a lower speed. Set this speed as the minimum speed *v*^min^. When the density is greater than the upper limit of density with no congestion, and smaller than the congestion density, according to the literature, it can be seen that the speed is related to the traffic flow density at this time, which can be expressed by Eq. (8).

(8) \begin{eqnarray*}{v}^{\min \nolimits }+({v}^{free}-{v}^{\min \nolimits })\cdot { \left[ 1-( \frac{\rho \left[ j \right] -{\rho }^{\min \nolimits }}{{\rho }^{jam}-{\rho }^{\min \nolimits }} )^{\alpha } \right] }^{\beta }\end{eqnarray*}

To sum up, the expression of the average inflow speed can be obtained, as shown in Eq. (9).

(9) \begin{eqnarray*}\begin{array}{@{}l@{}} \displaystyle v[j]= \left\{ \begin{array}{@{}l@{}} \displaystyle {v}^{free},\rho [j]\lt {\rho }^{\min \nolimits }\\ \displaystyle {v}^{\min \nolimits }+({v}^{free}-{v}^{\min \nolimits })\cdot { \left[ 1-( \frac{\rho \left[ j \right] -{\rho }^{\min \nolimits }}{{\rho }^{jam}-{\rho }^{\min \nolimits }} )^{\alpha } \right] }^{\beta },\rho [j]\in [{\rho }^{\min \nolimits },{\rho }^{jam}]\\ \displaystyle {v}^{\min \nolimits },\rho [j]\gt {\rho }^{jam} \end{array} \right. \\ \displaystyle \end{array}\end{eqnarray*}

*α* and *β* are the specified parameters which should be set in advance. *ρ*[*j*] is the traffic density from the upstream section to the end of the queue in the downstream, which can be calculated according to formula (10).

(10) \begin{eqnarray*}\rho [j]= \frac{N[j]-x[j]}{n(l- \frac{x[j]}{n\cdot {\rho }^{jam}} )} .\end{eqnarray*}

*N*[*j*] − *x*[*j*] represents the number of vehicles from the upstream section to the end of the downstream queue. $l- \frac{x[j]}{n\cdot {\rho }^{jam}} $ represents the queue length between the upstream sections and downstream sections.

#### Delay model for oversaturated arterial

The total delay of the oversaturated arterial can directly reflect the performance of traffic signal control. Therefore, the delay model is constructed by module of offset optimization with the optimization objective of total delay minimization. The minimum delay optimization model in each cycle can be expressed as Eq. (11).

(11) \begin{eqnarray*}minD\mathit{ = }min\sum _{e\mathit{=}1}^{I}\sum _{i\mathit{=}1}^{P}{d}_{ij}^{e}.\end{eqnarray*}

The constraints contain equations from Eqs. (4.1) to (4.10). In addition, several offset relationship needs to be provided to constraint the optimization objective. These constraints are shown in Eqs. (12)–(14).

(12) \begin{eqnarray*}& & -{c}_{cycle}\lt {O}_{j}^{e,e+1}\lt {c}_{cycle}\end{eqnarray*}

(13) \begin{eqnarray*}& & -{c}_{cycle}\lt {O}_{j}^{e+1,e}\lt {c}_{cycle}\end{eqnarray*}

(14) \begin{eqnarray*}& & {O}_{j}^{e,e+1}+{O}_{j}^{e+1,e}={c}_{cycle}\end{eqnarray*}

(15) \begin{eqnarray*}& & t{g}_{min}\leq {G}_{i}^{e}\leq t{g}_{max}\end{eqnarray*}

*minD* represents the optimization objective of minimizing total delay of oversaturated arterial, which is the sum of the delays corresponding to all phases for all intersections in each cycle. Formula (12) and formula (13) represent the range that offset needs to meet. Equation (14) represents the offset relationship between the upstream and downstream intersection. In this paper, the upstream direction and downstream direction along oversaturated arterial belong to the same coordination signal group. Therefore, the sum of these two offset is equal to a cycle time. Equation (15) represents the green time for each phase at all intersections along oversaturated arterial should be restricted to the range of $ \left[ t{g}_{min},t{g}_{max} \right] $.

#### Optimization algorithm

**Figure 6:**
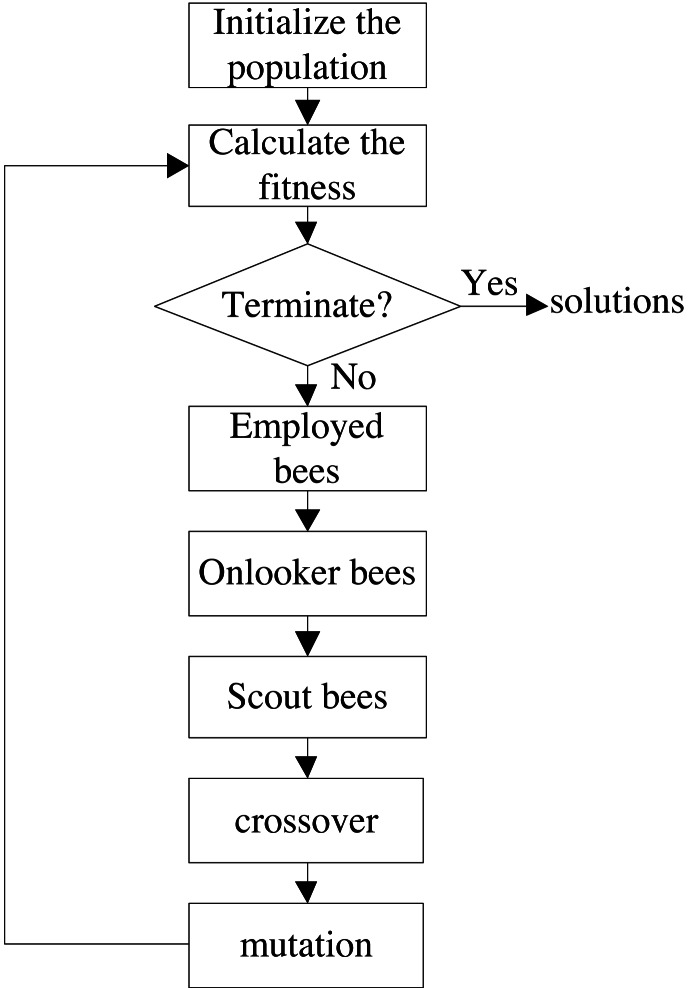
The flowchart of the IGAABC.

In order to obtain better solutions, an improved hybrid artificial bee colony algorithm IGAABC is used to solve the optimization model. The flowchart is shown in Fig. 6, and the details of IGAABC have been given in our previous paper (Xu, Chen & Xu, 2019).

### Arterial spillover detection

**Figure 7:**
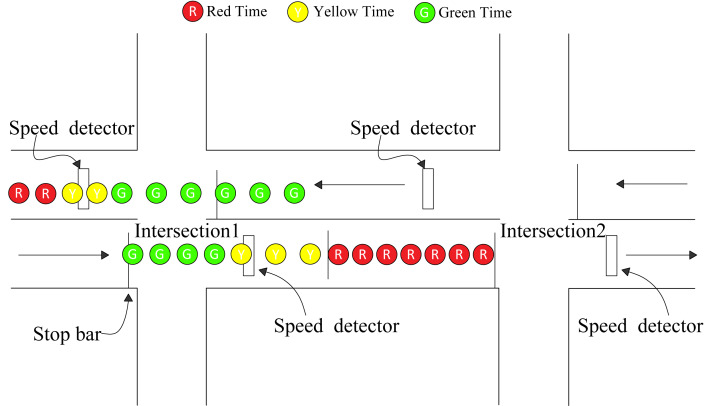
Arterial spillover detection.

The basic coordination control parameters of green time plans and offset can be obtained by the intersection coordination control method and offset optimization module, which can be regarded as the input parameters to control the simulated oversaturated arterial. Afterwards, the corresponding detection will detect possible queue overflows of each section along oversaturated arterial in real time. Assume that the east–west direction is the coordination direction of arterial. The upstream direction and downstream direction along oversaturated arterial are set as the same signal group. Deploy the necessary detectors and apply the queue spillover identification method (Xu, Chen & Xu, 2019) to identify potential queue spillover for each section along oversaturated arterial. As shown in Fig. 7, either the upstream or downstream direction along oversaturated arterial is detected a potential queue spillover, the green time of coordination phase will be cut off. The spillover detection method is the same for all intersections along oversaturated arterial.

### Arterial spillover management

The remaining green time will not be returned by the queue spillover control method to the coordination signal group until queue dissipation is detected at both the upstream and downstream directions of the coordination phase. The queue dissipation identification method and queue spillover control method is provided in our previous article (Xu, Chen & Xu, 2019). If two phases belong to the same signal group, the module of arterial spillover management will implement queuing overflow control method if the queue spillover of any sections controlled by the same signal group is detected. The remaining green time will not be returned to the coordination phase until all the sections belonging to the same signal light group are detected queue dissipation.

## Case Study

### Simulation settings

**Figure 8:**
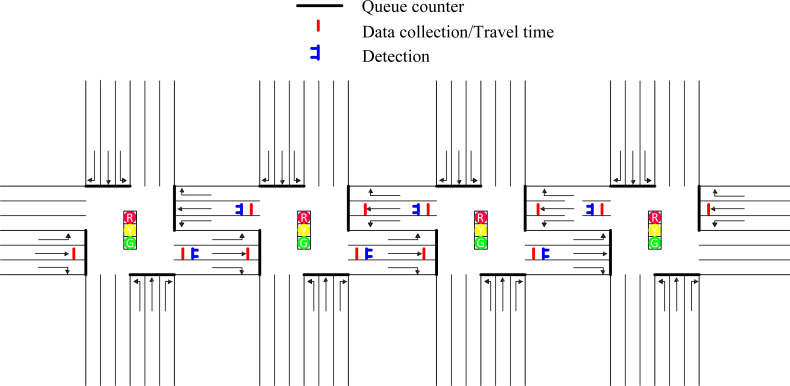
Deployment diagram for detectors along oversaturated arterials.

At the beginning of each cycle, real-time traffic flow information should be provided as input to traffic coordination control method to optimize the coordination parameters of green time plans and offset for all intersections along arterial. Therefore, it is necessary to configure the necessary detectors on the oversaturated arterial. Figure 8 is the deployment diagram for detectors along oversaturated arterial.

 Activate the options of link evaluation for all links on the software of VISSIM4.3. Traffic flow information such as queue length and number of vehicles for all links can be directly collected by this configuration, without the need to set a specific detector.

Queue counters numbered from 1 to 16, which are deployed near the parking lines of each intersection, can be applied to detect the queue length and the number of vehicles of each link at the beginning of each cycle. As shown in Fig. 8, the solid black line near the parking line at the intersection represents the queuing counter deployed on each link.

The data collection point and the travel time detector can obtain the number and time of vehicles passing through the detector, respectively. The required deployment position and the number of these two kinds of detectors are the same. Therefore, both these two types of detectors are indicated by a solid red line in Fig. 8. Considering that the capacity of the link from which vehicles are leaving the oversaturated arterial is large enough, the detectors will not be deployed on these links. Performing relevant calculations according to the detected real-time data, the average acceleration of the vehicle passing through a certain road segment can be obtained, which in turn provides a basis for the arterial spillover detection module.

The solid blue line, as shown in Fig. 8 represents the vehicle detector. The speed of the vehicle passing the detector can be detected by the deployed six detectors. The possible queue spillover of link 1-2, link 2-1, link 2-3, link 3-2, link 3-4 and link 4-3 can be identified and controlled promptly according to the computation results based on the collected traffic information.

**Figure 9:**
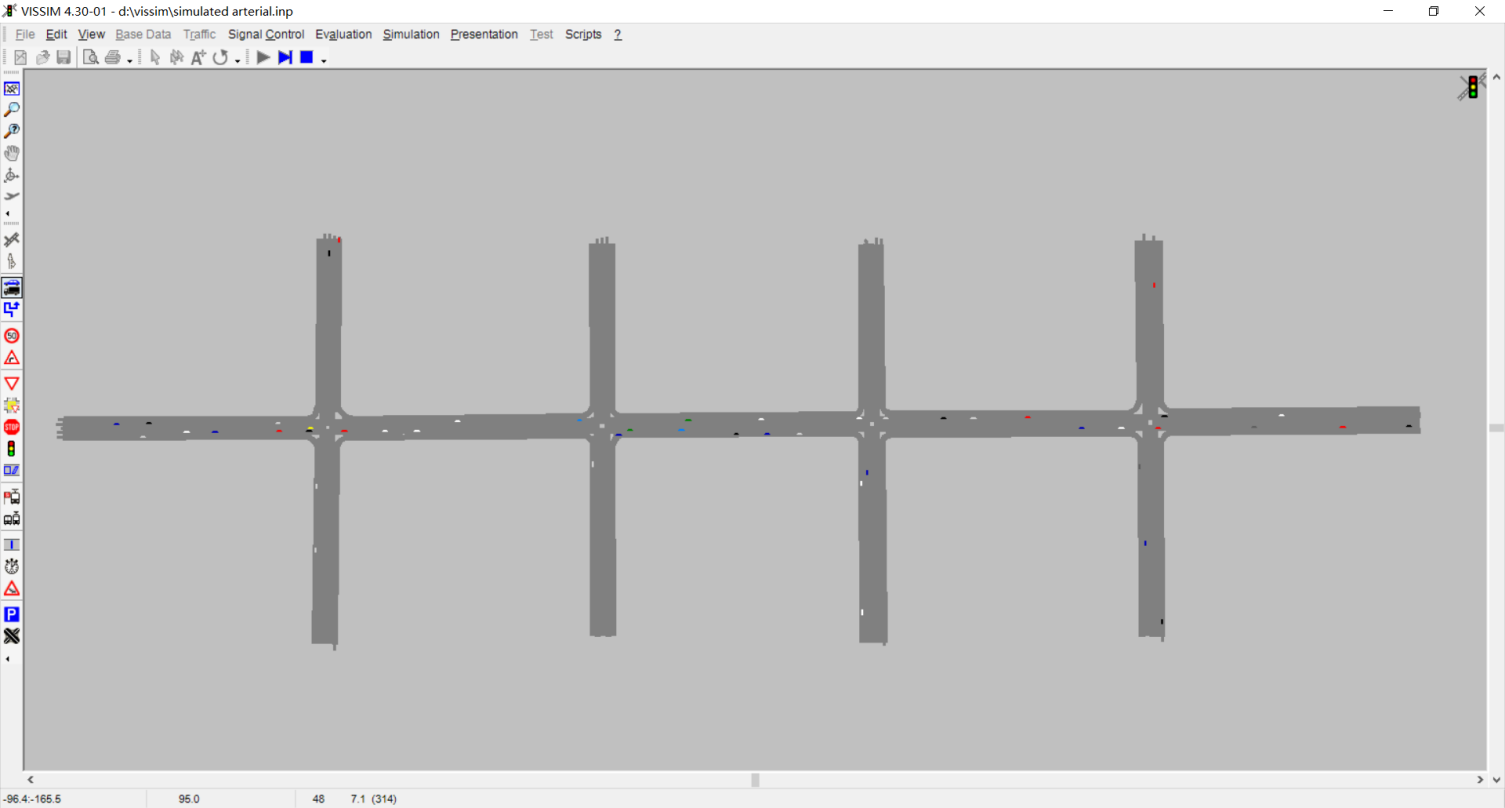
Arterial in VISSIM4.3.

In this paper, the proposed traffic coordination control method is evaluated on an oversaturated arterial with four intersections. Figure 9 shows the simulated road network of arterial drawn by VISSIM4.3 in this paper. The total simulation time of the road network is set as one hour. The hot start time is set as eight signal cycles. It means that we can collect the results of simulation evaluation after eight cycles. We will introduce the relevant parameters and road network configuration next.

As shown in Fig. 9, the studied arterial is composed of four intersections. The intersections along arterial are numbered from one to four, which is denoted by intersection 1, intersection 2, intersection 3, intersection 4. All links in the simulated arterial include three lanes where lane 1, lane 2 and lane 3 numbered from left to right on each link represent left-turn lane, through lane and right-turn lane, respectively. In this section, the software of VISSIM 4.3 is applied to simulate traffic behavior along oversaturated arterials to evaluate the performance of different traffic coordination methods. Python provides the coordination parameters of green time plans and offset optimized by different traffic coordination control method to VISSIM4.3. The number of signal groups of each intersection is set as three. The right-turn behavior of all intersections is not controlled by traffic signal. Therefore, the right turn phase signal group is not configured. The left-turn phase along the east–west direction is set as signal group one denoted by SG1. All through and left-turn phases along the north-south direction along oversaturated arterial are set as signal group two denoted by SG2. The phase of east–west through is set as the third signal group denoted by SG3. SG3 is set as the coordination phase along oversaturated arterial in this section.

Assume that the saturation flow rate ${s}_{i}^{e}$ for all links is the same, which can be set as 2000 vehicle/h. The length of all links is the same, which can be set as 200 m. Minimum green time *tg*_min_ andmaximum green time *tg*_*max*_ for all phase at each intersection are set as 10 s and 50 s, respectively. The width of the intersection is set as 40 m. *c*_*cycle*_ is set as 140 s. The simulation evaluation results are based on the data obtained at the predicted level of *P*_*h*_ = 5. The initial offset for all intersections is set as 0. The yellow time is set as 3 s uniformly. The parameter in the inflow speed calculation is set as *α* = *β* = 0.5. The initial green time allocation plan for each intersection is shown in Table 1.

**Table 1:** Initial green time allocation plan for each intersection.

	Intersection1	Intersection 2	Intersection 3	Intersection 4
SG1	35	45	45	45
SG2	65	55	55	65
SG3	40	40	40	30

In order to obtain simulation results of different traffic signal control methods, traffic flow information at different entrances should be provided. Table 2 shows the traffic flow information at the entrance of each intersection during the simulation period. Among them, NB, SB, EB and WB represent the north, south, east and west of the intersection, respectively. Entrance of L, T, and R indicate left, straight, and right turns, respectively. Total represents total traffic flow. The unit is vehicle / h.

**Table 2:** Traffic flow for all entrance at each intersection.

	Entrance	L	T	R	Total
Intersection 1	NB	147	256	166	569
	WB	175	963	183	1,321
	SB	181	221	168	570
	EB	0	0	0	0
Intersection 2	NB	270	300	105	675
	WB	0	0	0	0
	SB	285	510	60	855
	EB	0	0	0	0
Intersection 3	NB	4	120	15	135
	WB	0	0	0	0
	SB	0	0	0	0
	EB	0	0	0	0
Intersection 4	NB	476	0	280	756
	WB	0	0	0	0
	SB	0	0	0	0
	EB	0	1,820	336	2,156

The parameters of the optimization algorithm IGAABC, applied to solve offset optimization and intersection coordination control in this paper, are set as follows: the parameter of *cst* isset as 100. The parameter of *bee* is set as 20. *p*_*co*_ is set as 0.5. The mutation probability *p*_*m*_ is set as 0.01. The number of iterations is set as 100.

In this section, the methods proposed in this paper will be compared with the existing methods based on the simulation evaluation results of the delay for each phase, the average number of cars stopped, and the maximum queue length to verify the effectiveness of our proposed method.

### Simulation results

In this section, the performance index of delay, stop times and queuing length will be collected by VISSIM4.3 to evaluate the control effect of the arterial coordination control method proposed in this paper. The method proposed in this paper will be compared with the delay minimization method and traffic coordination control method without offset optimization. The description of these three algorithms is introduced as below.

(1) Method 1: The coordination control method without offset optimization, which applies intersection coordination control method (Xu, Chen & Xu, 2019) alone to optimize the green time scheme for all intersections along oversaturated arterial.

(2) Method 2: the delay minimization method, a novel minimization delay algorithm (Li, Huang & Lo, 2018) that analyzes the relationship between offset and delay model, which take the remaining queue into account to improve the classical minimization delay model.

(3) Method 3: The proposed two-way delay minimization method in this paper, which considers the impact of traffic density on vehicle speed and predicts the arriving rate of vehicles to adapt to evolving traffic demand.

In order to guarantee the validity of the simulation evaluation results, simulation experiment has been conducted five times under the same conditions to obtain the average value of the simulation results. Table 3 shows the simulation results of total vehicle average delay for all intersections along oversaturated arterial with different methods.

**Table 3:** Comparison of total vehicle average delay with different methods.

	Method 1	Method 2	Method 3
Intersection 1	446.9	426.8	418
Intersection 2	292.8	294.7	275.7
Intersection 3	55.2	54.1	54.4
Intersection 4	360.4	280.2	250.6

As can be seen from Table 3, the total delay of the whole oversaturated arterial with different methods is 1,155.3, 1,055.8 and 998.7, respectively. Compared with the method 1, the method 3 proposed in this article can decrease the total delay by 13.6%. Compared with the method 2, the method 3 proposed in this article can decrease the delay by 5.4%. Comparing the delay of vehicles at these four intersections, it can be seen that the delay of intersection 3 is almost same. The delay of vehicles at intersections 1, 2, and 4 is decreased by 6.5%, 5.8%, and 30.5%, respectively compared with the method 1. Compared with Method 2, the delay of vehicles at intersections 1, 2, and 4 is decreased by 2.1%, 6.4%, and 10.6%, respectively. The total average vehicle delay can be decreased much compared with the method 1 and method 2.

One of the major purposes of arterial coordination control method research is to improve the traffic along oversaturated arterial. The performance parameters along the upstream and downstream directions of arterial are the key indicators which can reflect the coordination control effect along arterial. It is assumed that the downstream direction along arterial is from west to east, which can be expressed as WE; the upstream direction arterial is from east to west, which can be expressed as EW. By analyzing the average delay (delay) and the average number of stops (stops) along the upstream and downstream directions of arterial, the simulation results can be obtained as shown in Table 4.

**Table 4:** Comparison of average delay and number of stops with different methods.

	Direction	Performance Index	Method 1	Method 2	Method 2
Intersection 1	WE	delay	58.7	50.5	46.2
		stops	4.75	4.68	4.61
	EW	delay	149.5	142.8	141.7
		stops	14.01	14.11	13.01
Intersection 2	WE	delay	34.6	31.6	27.7
		stops	0.95	0.92	0.83
	EW	delay	14.2	18.6	12
		stops	0.44	0.45	0.41
Intersection 3	WE	delay	1.9	0.9	0.8
		stops	0.24	0.19	0.15
	EW	delay	49.1	49.5	49.3
		stops	1.89	1.97	1.92
Intersection 4	WE	delay	67.8	66	53
		stops	0.13	0.13	0.11
	EW	delay	60.9	60.2	45.7
		stops	5.68	5.43	4.57

It can be seen from Table 4 that the variation trend of average number of stops along oversaturated arterial is consistent with the delay of the vehicles. Therefore, either of both these performance index can be selected as measure to evaluate the control effect of different methods. In this paper, the average vehicle delay along oversaturated arterial is regarded as the research object to conduct the data comparison and analysis. The vehicles delay with the method 3 proposed in this article along the downstream direction of oversaturated arterial at intersections 1, 2, 3, and 4 is decreased by 8.5%, 12.3%, 11.1%, and 19.7%, respectively, compared with the method 2. The delay along the upstream direction of oversaturated arterial is decreased by 0.8%, 35.5%, 0.4% and 24.1%, respectively. Compared with the method 1, the delay along the downstream direction of oversaturated arterial at intersections 1, 2, 3, and 4 is decreased by 21.3%, 19.9%, 57.9%, and 21.8%, respectively. The delay along the upstream direction of oversaturated arterial at intersections 1, 2, 3, and 4 is decreased by 5.2%, 15.5%, 0, and 25%, respectively. Considering that method 3 can optimize the two-way delay and take into consideration the influence of flow density on vehicle speed, the proposed model is more accurate. This method can decrease the average vehicle delay along the upstream and downstream directions of oversaturated arterial in theory. The simulation results obtained by the actual simulation evaluation verify the effectiveness of the proposed method in decreasing delay.

Under the oversaturated traffic condition, the queue length can reflect the effect of the traffic control to a certain extent. If the queuing length of each section is small and relatively balanced, it indicates that the control effect is good. Table 5 shows the simulation results of average queue length obtained by simulation evaluation. Assume WE, EW, SN, NS represent the queue length of the intersection of east–west direction, east–west direction, south-north direction and north-south direction, respectively. The unit is meter.

**Table 5:** Comparison of average queue length with different methods.

	Direction	Method 1	Method 2	Method 3
Intersection 1	WE	177	144	134
	EW	10	16	14
	SN	18	17	16
	NS	20	19	19
Intersection 2	WE	146	136	121
	EW	17	16	15
	SN	91	89	83
	NS	171	171	106
Intersection 3	WE	19	11	15
	EW	138	126	115
	SN	97	95	84
	NS	103	97	78
Intersection 4	WE	36	23	21
	EW	184	179	119
	SN	7	4	13
	NS	164	164	107

**Figure 10:**
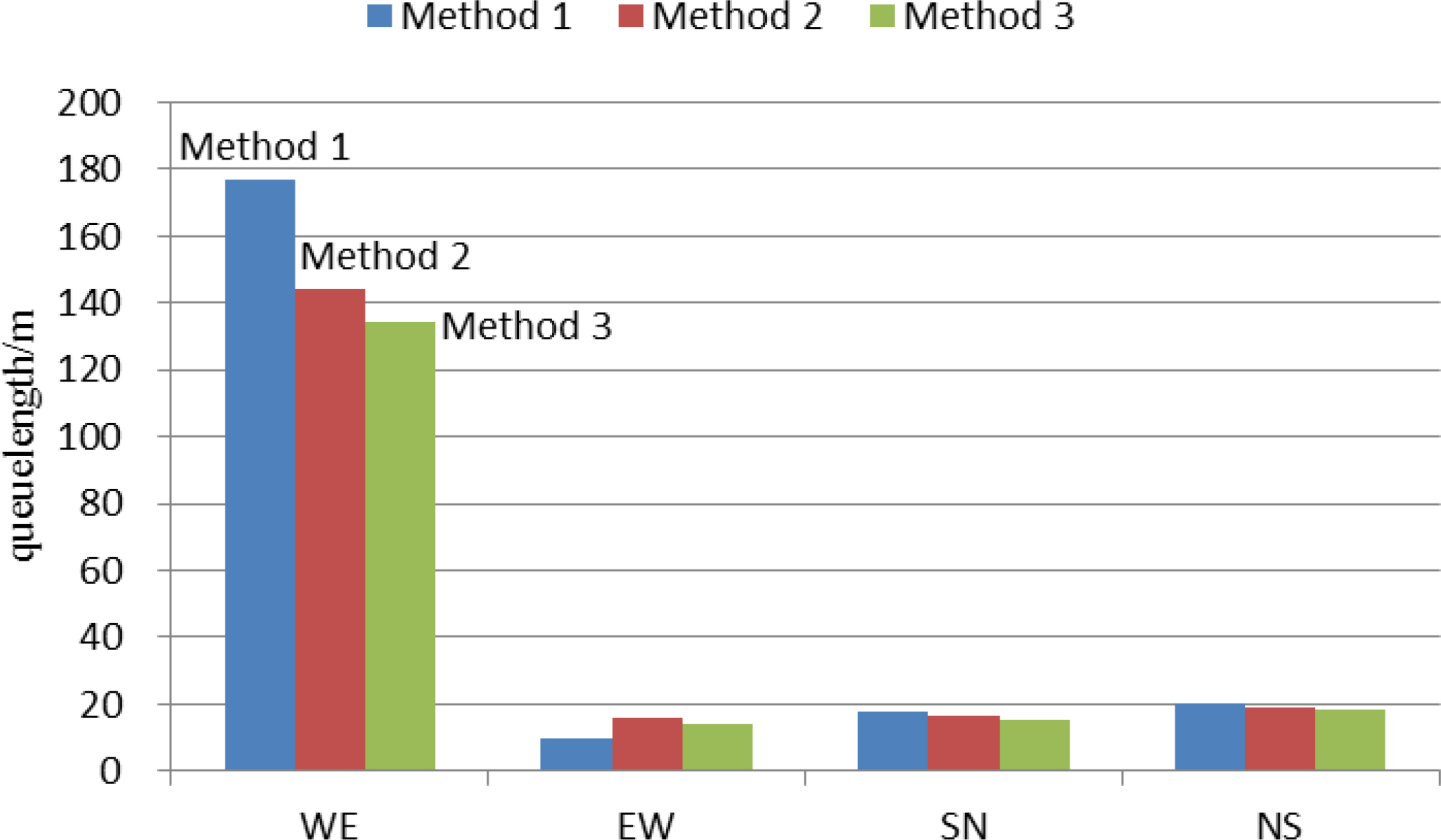
Comparison of average queue length with different methods at intersection 1.

It can be seen from Table 5 that the queue length obtained by the method 3 is relatively small. In addition, the queue length of each link along oversaturated arterial is relatively balanced. In order to show the control effects of these three traffic signal control methods intuitively, we take intersection 1 as an example to analyze the queue length in this paper. The results are shown in Fig. 10. It can be seen from Fig. 10 that the control effect of method 3 is optimal.

It takes some time to collect traffic flow information and optimize the coordination parameters for our proposed method. By sampling and analyzing the algorithm optimization time for each cycle, the results are as follows: the maximum optimization time is 91.23 s, and the average optimization time is 84.74 s. The signal period is 140 s. Therefore, the proposed method in this paper can duly obtain the optimization results within a signal cycle time, which meet time requirements of arterial coordination control.

### Sensitivity analysis

In order to evaluate the control effect of arterial coordination control method proposed in this paper under different traffic flow and turning behavior conditions, this paper analyzes the sensitivity of the proposed method for both these aspects. Similarly, the total vehicle average delay along arterial are selected as the basis for evaluation. The traffic flow coefficient m can be set as 0.2, 0.4, 0.6, 0.8, 1.0 and 1.2 to get the total vehicle average delay in six different scenarios, as shown in Table 6.

**Table 6:** Comparison of total vehicle average delay in six different scenarios.

m	Method 1	Method 2	Method 3
0.2	710.5	714.3	708.7
0.4	816.7	815.2	809.3
0.6	817.4	817.6	814.5
0.8	910.7	925.8	905.4
1	1,155.3	1,055.8	998.7
1.2	1,756.3	1,596.4	1,232.6

As can be seen from Table 6, although the control effect of the method 3 is not obvious when the traffic volume is low, the total vehicle average delay can be decreased much by the Method 3 with the increase of traffic volume.

To conduct the sensitivity analysis of turning behavior, three turning ratios are set in this paper. Suppose β=[L, T, R], where L, T, R represent the ratio of left turn, straight travel and right turn, respectively. As shown in Table 7, the method 3 can achieve better control effect under different turning rates, which validates the control effect of our proposed arterial coordination control method.

**Table 7:** Comparison of total vehicle average delay with different turning behavior.

β	[1,1,1]	[1,2,1]	[2,1,1]
Method 1	1,547.6	1,257.8	1,392.1
Method 2	1,159.3	1,152.3	1,248.6
Method 3	981.7	952.4	954.6

## Conclusion and future outlook

This paper proposes a traffic coordination control method to optimize the coordination parameters of green time plans and offset for all intersections along oversaturated arterial. In order to adapt to evolving traffic demand, the model predictive control method is applied to predict the arriving rate of vehicles. To deal with the problem that vehicle speed will change with traffic density, we propose an improved delay minimization method, which takes into consideration the impact of traffic density on vehicle speed to optimize the coordination parameters of offset for all intersections. In order to reduce the computation time of coordination parameter optimization to apply the proposed method to complicated area road network, the optimization algorithm of IGAGBC can be further improved in the future.

Good traffic coordination methods can improve traffic efficiency and can only increase people’s income indirectly. If one wants to really increase people’s income, you still need government policy help. The government can rationally reduce the city’s parking fees and management, speed up the construction of road traffic infrastructure and increase the carrying capacity, increase priority to develop public transportation and encourage public transportation, and increase fiscal expenditures and reduce road tolls.

##  Supplemental Information

10.7717/peerj-cs.319/supp-1 Supplemental Information 1 Code

10.7717/peerj-cs.319/supp-2 Supplemental Information 2 Simulation evaluation data

## Additional Information and Declarations

### Data Availability

The following information was supplied regarding data availability: The raw measurements and code are available in the Supplemental Files.
